# Evaluation of the compostability and toxic effects on *Hordeum vulgare* and *Cynodon dactylon* plants of plastic bags manufactured with compostable plastics

**DOI:** 10.1371/journal.pone.0318938

**Published:** 2025-03-25

**Authors:** Erik Saúl Huidobro-Medina, María Neftalí Rojas-Valencia

**Affiliations:** National Autonomous University of Mexico, Institute of Engineering, School Circuit S/N, University City, Coyoacán, Mexico City, Mexico; Khalifa University, UNITED ARAB EMIRATES

## Abstract

Because of the problem related to the accumulation of plastic bags in the environment, polymers called “bioplastics” have been developed. These materials are designed to reintegrate quickly into the environment, reducing thus environmental damage. However, when testing disposable bags made with biopolymers using standardized methodologies, it has not been possible to observe that they satisfactorily meet the criteria to be considered compostable. The present study evaluated compliance with the compostability criteria according to international standards established by the International Organization for Standardization (ISO) of three certified compostable plastic bags (BioW, Comph and Ecol) manufactured with polybutylene adipate-co-terephthalate, polylactic acid and corn starch, performing chemical composition tests using attenuated total reflectance Fourier Transform infrared (ATR-FTIR) spectroscopy and inductively coupled plasma – optical emission spectroscopy (ICP-OES), and disintegration, biodegradation and ecotoxicity tests. The results showed that the three bags contain the polymers reported in their data sheet and possibly polyethylene. Likewise, all of them comply with the maximum permissible limit of metals. Comph and Ecol met the disintegration criteria. No bag met the aerobic biodegradation criterion, with Ecol showing the highest percentage (51%) and Comph the lowest (3%). None of the bags had toxic effects on plants and neither met the biodegradation criterion necessary to be considered 100% compostable.

## 1. Introduction

Since the industrial revolution, advances in the development of technologies for the synthesis of materials have been of great relevance to the world economy and these developments have allowed various industries to adapt the same materials to manufacture very different products [1].

A very common use for plastics is the manufacture of packaging and/or disposable bags for transporting food, medicines, tools, among many other items. These packages and bags have been manufactured mainly from polymers such as polyethylene (PE) and polypropylene (PP), conventional plastics synthesized from petroleum, characterized by their resistance and versatility, in addition to their low cost, which has allowed them to replace other materials [2]. Some estimates indicate that between 500 billion and one trillion plastic bags are consumed worldwide annually [3]. And according to Ritchie [4], in 2019, the packing and/or packaging manufacturing industry was the one that consumed the most plastics worldwide, with approximately 42% of the total production.

Tons of packaging and single-use plastic bags are discarded daily, with a minimum percentage being recovered and recycled, while a significant amount is not treated, ending up in soils, bodies of water and, in the best of the cases, landfills. This has led to their accumulation in the environment where they damage the biodiversity, health, food chain and landscape, resulting in one of the most serious problems of our time [5].

One of the various solutions proposed to control the environmental damage caused by plastic bags is the replacement of the conventional plastics with which they are commonly manufactured (PE and PP) with bioplastics. Said bioplastics are mainly produced from mostly renewable sources [6]. These materials are expected to perform well in the biodegradation process once they are in the environment, allowing their assimilation into natural cycles and eliminating the problem of environmental accumulation [7].

The bioplastic bags (BP) industry has grown in recent years seeking to find the appropriate biopolymer to better replace conventional plastics, without losing the benefits they have brought to the industry. For this purpose, different materials such as Polybutylene Adipate-co-Terephthalate (PBAT), Polylactic acid (PLA), Poly butylene succinate (PBS), Polyhydroxyalkanoates (PHA), Polycaprolactone (PCL), starches, alginic acid derivatives, among others, have been developed. However, during this search and development of biodegradable and compostable polymers, some companies have marketed BP considered to be biodegradable or compostable that, when tested, have not shown these processes, or have shown them to a very limited extent, a situation considered as “Green washing” that does not offer a solution to the environmental pollution problem [8].

Likewise, it is important to highlight that some studies have shown that some BPs can have harmful or toxic effects on different organisms such as fish and insects exposed to them, either in large pieces or in the form of microplastics [9–11].This new problem is a global challenge that society has sought to address by establishing, standardizing, and regulating different methods of evaluating the disintegration, aerobic or anaerobic biodegradability, chemical composition and ecotoxicity, and thus be able to certify products that do comply [12]. However, it seems necessary to improve the regulations and methodologies that set up the guidelines for this industry and the products it develops [13].

Therefore, using standardized international methodologies, the present study evaluated the disintegration, aerobic biodegradability, chemical composition and ecotoxicity of three different plastic bags certified as compostable manufactured with a mixture of PBAT, PLA and corn starch, marketed in Mexico, in order to verify their compliance with the compostability criteria established by the International Organization for Standardization and Organisation for Economic Co-operation and Development.

One of the main contributions of this research lies in questioning current certifications. The bags analyzed have certifications that endorse their compostability; the results obtained using standardized methodologies reveal that none of these materials meet the biodegradation criteria necessary to be considered 100% compostable. This finding is very significant, as it challenges the manufacturers’ claims and shows the real compostability of the products under controlled conditions. It is important to mention that many consumers currently consider these certifications as reliable indicators of environmental safety.

Furthermore, the approach taken in this research seeks to be critical and evidence-based in order to encourage the development of stricter and more practical standards that had better align compostability expectations with the reality of products.

It was also possible to show that improvements are needed in the field of product design made from this type of material. In particular, the low level of biodegradation observed, which in the best case reached only 50.96%, indicates that current bag formulations and manufacturing processes need to be optimized to ensure complete decomposition under real composting conditions.

On the other hand, from a regulatory perspective, the study provides a basis for proposing changes to international standards governing the certification of compostable products, by demonstrating that current methodologies may not be sufficiently rigorous. Finally, products based on compostable polymers are usually more expensive than conventional plastics, and their marketing is strongly linked to their environmentally friendly characteristics. For this reason, it is very important that they fully and truly comply with the established standards, not only to protect the environment, but also to safeguard the economic viability of this emerging industry.

## 2. Materials and methods

This study was divided into the following stages: 1) Acquisition of the certified compostable bags to be tested, 2) Analysis of the chemical composition of the acquired bags using attenuated total reflectance Fourier Transform infrared (ATR-FTIR) spectroscopy and inductively coupled plasma – optical emission spectroscopy (ICP-OES), 3) Evaluation of the disintegration, aerobic biodegradation and ecotoxicity of compostable bags in accordance with *ISO 20200:2015, ISO* [14] *14855:2018-2* [15] standard and *OECD Test No. 208* [16], and 4) Statistical analysis.

### 2.1. Selection of the compostable plastic bags to be tested

Disintegration, aerobic biodegradation and ecotoxicity tests were carried out on three compostable bags (BioW, Comph, and Ecol). The selection criteria were based on easy accessibility, components similarity and compostability certificate. Table 1 shows the percentage composition reported in the data sheet of the selected bags.

**Table 1:** Percentage composition reported in the data sheet of the selected compostable bags.

Component	BAG		
	BioW (15)	Comph [15]	Ecol [15]
PBAT	50%	50–70%	N/I
PLA	15%	5–15%	N/I
Corn starch	35%	25–35%	N/I

BPI certification in compostable by American Society for Testing and Materials (ASTM).

PLA, polylactic acid; PBAT, polybutylene adipate-co-terephthalate; N/I, no information.

### 2.2. Analysis of the chemical composition of the bags

The analysis of the chemical composition of the bags was carried out using ATR-FTIR spectroscopy techniques for polymers and ICP-OES spectrometry for metals.

### 2.3. Polymer analysis through ATR-FTIR spectroscopy

The infrared spectra of each compostable bag were obtained by placing a small square of approximately 1.0 x 1.0 cm on the diamond crystal of the Thermo Scientific NICOLET 6700 FT-IR spectrometer, adjusting the screw, and proceeding to read the spectrum. The infrared spectrum reading of each compostable bag was carried out 10 times.

### 2.4. Metal analysis through ICP-OES spectrometry

In order to carry out the quantification of metals, a digestion of the compostable plastic bags was previously performed. A Milestone microwave oven model Ethos Easy and the following reagents and materials were used: compostable plastic bag samples in pieces of 1 to 3 mm, 30% hydrogen peroxide, 70% nitric acid, acidic water (2%), filter paper, funnels, 50 mL volumetric flask and Teflon digestion tubes.

0.500 g of each compostable bag were weighed on an analytical balance. Subsequently they were placed inside the Teflon tubes, 2 mL of hydrogen peroxide and 6 mL of 70% nitric acid were added. Likewise, a blank tube was prepared in which only the reagents were added without plastic sample. A tube with the reagents and a standard metal solution was prepared as control. The Teflon tubes were closed and then introduced into the oven. The microwave oven was programmed to reach the temperature of 200 °C in 20 minutes and then maintain the temperature at 200 °C for 15 minutes. Once the cycle was completed, the tubes were allowed to reach room temperature.

The tubes were removed from the oven and placed according to their numbering in a wooden rack. Subsequently, the content (digested plastic) was extracted by pouring it into a funnel with filter paper attached to a 50 mL volumetric flask. The Teflon tubes were then washed with a small amount of acidic water and the wash water was emptied into the volumetric flask. Once all the liquid generated in the digestion was in the flasks, a small washing of the filter paper and funnel was carried out with acidic water, and each flask was filled to 50 mL with acidic water, then stoppered and the content was mixed to homogenize it. Finally, the 50 mL of the volumetric flasks were emptied into plastic bottles labeled with the corresponding sample and kept until they were read in the Agilent Technologies 5100 ICP-OES spectrometer. The digestion of each plastic sample was carried out in triplicate.

The metals evaluated in the plastic bags were arsenic, cadmium, cobalt, copper, chromium, molybdenum, nickel, lead, selenium, and zinc, which were compared with the maximum permissible limits (MPL) established in *UNE EN 13432:2001. Containers and packaging. Requirements for containers and packaging that can be recovered through composting and biodegradation. Test program and evaluation criteria for final acceptance of the container or packaging* [17]; *ASTM D 6400. Labeling of plastics designed to be aerobically composted in municipal or industrial plants* [18] and *NOM-004-SEMARNAT-2002* standards. *Environmental Protection. Sludge and biosolids. Specifications and maximum permissible limits of contaminants for use and final disposal* [19].

### 2.5. Disintegration, aerobic biodegradation and ecotoxicity tests of the compostable bags

The tests were carried out in accordance with the methodologies suggested in point 10 of *NMX-E-273-NYCE-2019* standard. *Plastic industry – Compostable plastics – Specifications and test methods*. The selection of the methodologies was made considering the ease of setting up the experiments and the acquisition of certain inputs and materials. The methodology used in each test is described hereinbelow.

### 2.6. Disintegration evaluation

The disintegration test was carried out following the methodology established by *ISO 20200:2015 standard* [14]. *Plastics-Determination of the degree of disintegration of plastic materials under simulated composting conditions in a laboratory scale test*. To carry out this test, the following materials were required: sawdust, rabbit food, mature compost with a high content of stabilized microorganisms from the CU, UNAM compost plant, corn starch, saccharose, corn oil and urea were used in the proportions indicated by *ISO 20200:2015* standard *[*14*]*. Likewise, 12 lidded polypropylene containers measuring 30 X 20 X 12 cm having two perforations at a height of about 6.5 cm on the short sides of the container to allow gas exchange were employed.

The synthetic waste was prepared by thoroughly mixing all the materials. Subsequently 1 kg of synthetic waste was placed in each polypropylene container and 550 g of purified water were added to obtain a 55% wet waste. Finally, 1 kg of synthetic waste was kept, and 15 g of the test bags cut into 2.5 X 2.5 cm squares were added. Control containers with a polypropylene bag were also included. The containers (bioreactors) were mixed again until a homogeneous compost with plastic was obtained. The lids of each bioreactor were closed, and finally they were placed in an incubator at 58 °C (±2 °C), this being the beginning of the thermophilic period mentioned in the standard. Three repetitions were performed per bag.

During the thermophilic period (incubation at 58 °C), the maintenance activities of the composting process specified in *ISO 20200:2015* standard [14] were carried out during the 54 days of the test. These activities were mainly the addition of water to compensate for evaporation, and the mixing of the synthetic waste from each bioreactor to prevent the generation of an anaerobic environment.

During the performance of the test, some qualitative indicators of adequate composting were monitored, these being: odor (ammonia-acid and odorless), visual appearance (yellow/light brown to dark brown or black), and mycelium development on the decomposing matter.

Once the 54 days of the test had elapsed, the completion of the test was prepared. The lids of all the bioreactors were opened, and the water contained in them was evaporated by keeping them at 58 °C. The drying process concluded once it was observed that each bioreactor was dry and at a constant weight for two days.

Once the bioreactors were dry and at a constant weight, the compost was extracted and sieved through 5 mm and 2 mm meshes. All the plastic material that did not pass through the sieve was recovered. Subsequently, the recovered plastic was washed with distilled water to remove adhered compost residues and dried at 40 °C until a constant weight was obtained. The last weight obtained from the plastic of each repetition was recorded.

Finally, to obtain the percentage of disintegration (D), the plastic material recovered was considered non-disintegrated material, and the material that passed through the sieves was considered disintegrated. The percentage of disintegration (D) was calculated using Equation 1:


$$D=\frac{mi-mr}{mi}*100$$ (1)

Where:

**mi** is the initial dry mass of the tested material.

**mr** is the dry mass of the material recovered after the test.

A validation methodology of the composting process was also carried out, which required measurements of dry mass and volatile solids of the synthetic waste before and after the composting process. The calculations of the decrease in volatile solids (R) of the composting process were carried out using Equation 2:


$$R=\frac{\left[m_{i}*\left(DM\right)i*\left(VS\right)i\right]-\left[m_{f}*\left(DM\right)f*\left(VS\right)f\right]}{\left[m_{i}*\left(DM\right)i*\left(VS\right)i\right]}*100$$ (2)

Where:

*R* is the percentage of decrease in volatile solids which must be equal to or greater than 30%.

**m**_***i***_ is the initial wet mass of synthetic waste placed in each reactor.

***(DM)i*** is the initial dry mass of synthetic waste expressed as a percentage and divided by 100.

***(VS)i*** is the content of volatile solids in the synthetic residue, expressed as a percentage and divided by 100.

**m*f*** is the final mass obtained at the end of composting (mass of wet compost).

***(DM)f*** is the final dry mass of compost, expressed as a percentage and divided by 100.

***(VS)f*** is the content of volatile solids in the compost, expressed as a percentage and divided by 100.

### 2.7. Evaluation of aerobic biodegradation

The method used to determine aerobic biodegradation was that established in *ISO 14855:2018-2* standard [15]. *Determination of the ultimate aerobic biodegradability of plastic materials under controlled composting conditions – Method by analysis of evolved carbon dioxide. Part 2: Gravimetric measurement of carbon dioxide evolved in a laboratory-scale test.*

For this test, the following reagents and materials were required: Soda lime in a size of 2 to 4 mm, anhydrous calcium chloride in a size of 2 to 3 mm, sodium hydroxide in previously crushed lentils in an approximate size of 2 to 3 mm, silica gel in 2 to 4 mm spheres, fine silica sand, 1 M sulfuric acid, methyl orange, TLC grade microcrystalline cellulose, compostable plastic test bags, homemade mature compost with rabbit compost no older than 3 months and commercial compost.

As for other materials and equipment, air flowmeters, acrylic columns, 500 mL, 250 mL, and 125 mL Erlenmeyer flasks with double-hole stoppers, laboratory hose, glass tube, an incubator with a thermostat and a capacity of 58 °C ±2 °C, alcohol thermometer with −20 to 150 °C range, constant air system, potentiometer, Shimadzu solids module carbon analyzer model SSM-5000A, muffle, oven, alumina-porcelain capsules and analytical balance with a sensitivity of 0.0001 g were required.

In accordance with *ISO 14855:2018-2* standard [15], this test is performed following different stages which are mentioned hereinafter: preparation of the inoculum, preparation of silica sand, preparation of the test material, start-up of the test procedure, measurement, and test completion.

The compost inoculum was prepared from a wet homemade compost and a wet commercial compost that were previously sieved through a 3 mm pore size mesh, later 60 g of homemade compost and 40 g of commercial compost were added in a 1 L beaker, then 10% of the total weight of distilled water was added and mixed. The preparation of the silica sand consisted of washing the material with running water until the water did not present turbidity, then the sand was washed with distilled water twice. Subsequently, the silica sand was placed in a glass container that was put in an oven at 105 °C to dry.

The preparation of the test material consisted mainly of fragmenting the plastic bags into small pieces of bag ranging from 1 to 2 mm and determining the total organic carbon (TOC) of the test bags and the microcrystalline cellulose (reference control material), which was carried out according to instructions of the manual of the Shimadzu carbon analyzer equipment model SSM-5000A.

**Fig 1:**
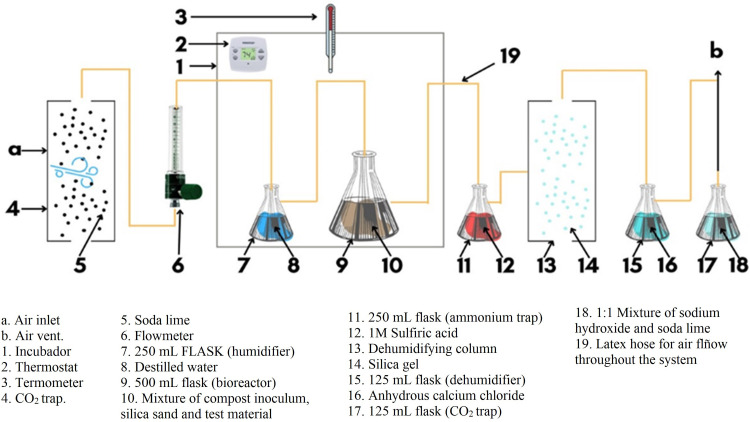
Diagram of the trap, bioreactor, humidifier, and dehumidifier system used in the test. Adapted from: ISO 14855:2018-2 [15].

**Fig 2:**
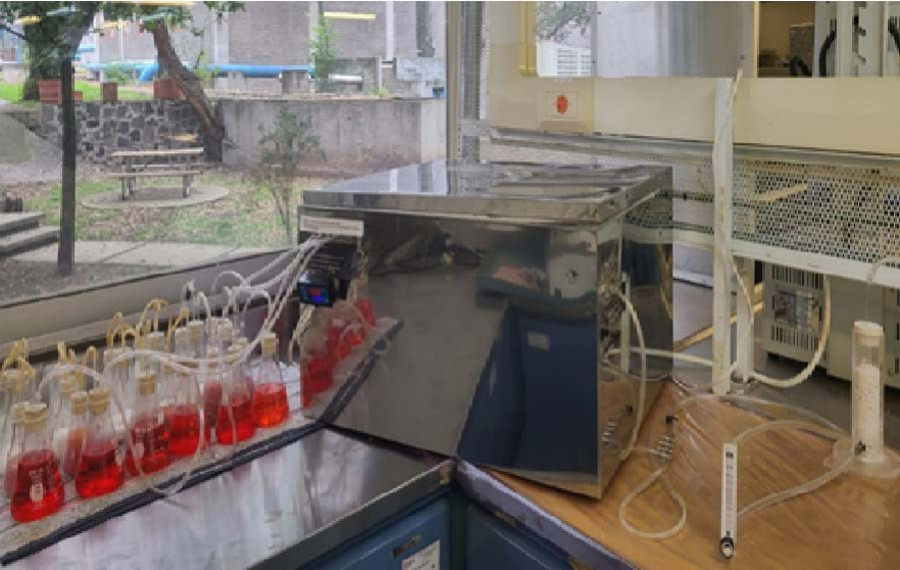
Complete image of the system assembled to carry out the biodegradation test.

The start-up of the test procedure began once the assembly of the system of bioreactors, humidifiers, dehumidifiers, and trap columns was completed. Fig 1 shows an example of the order in which each element was placed, and Fig 2 shows images of the actual assembly. Within the different containers that are part of the system, the following quantities of reagents were introduced: 100 g of soda lime in the first CO_2_ trap column, 150 mL of distilled water in the humidifier, 150 mL of H_2_SO_4_ in the ammonium trap, 100 g of silica gel in the dehumidifying column, 75 g of CaCL_2_ and 80 g of a 1:1 mixture of sodium hydroxide and soda lime in the exit CO_2_ trap.

**Fig 3:**
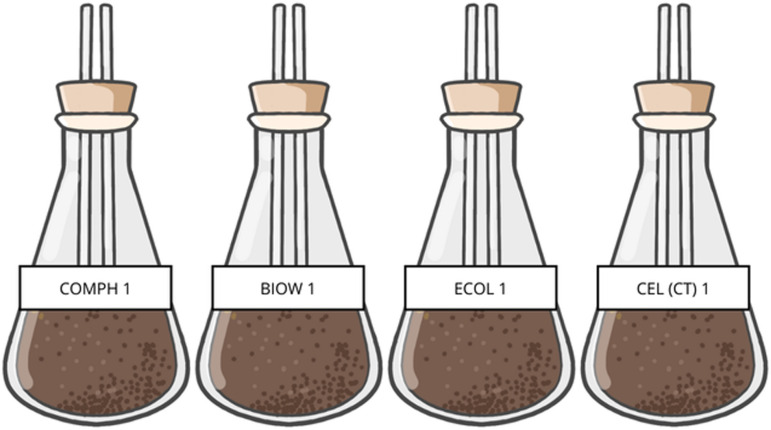
Bioreactors during their preparation, the plastic material used, and the cellulose can be seen on the left, in the middle the bioreactors with inoculum and silica sand, and on the right the weighing of a bioreactor.

The material for each bioreactor was prepared following *ISO 14855:2018-2* standard [15] procedure, using 1/3 of the quantities mentioned by said standard as a “typical case”. The quantities used are indicated hereinafter: 43.15 g (20 g of dry solids) of compost inoculum and 4.819 g of distilled water were added to bring humidity to 65% and the mixture was left to rest for 24 hours. Subsequently, 106 g of silica sand at 15% humidity (previously prepared with distilled water), 3.3 g of compostable test bag and 3.3 g of microcrystalline cellulose were added for control. Likewise, a blank bioreactor was prepared which consisted of only compost inoculum and silica sand. Finally, the mixture was adjusted at 90% humidity by adding 4.3 g of distilled water and the final mass was weighed. Fig 3 shows the preparation of the bioreactors.

Once the bioreactors were prepared and weighed, they were introduced into the incubator, they were connected to the air flow which was adjusted approximately between 10 mL/min and 30 mL/min. The incubator was then closed, and the temperature was adjusted at 58 °C ± 2 °C, which was confirmed with the thermometer.

The measurement of the amount of CO_2_ generated by the activity of the bioreactors was carried out by difference in weights (gravimetry) as indicated in the standard. The measurements were carried out daily until weight gain was no longer observed; thereafter the measurement was taken every other day for 43 days. As maintenance activities, the bioreactors were shaken every 7 days to avoid anaerobic conditions and to loosen the contents. Observations such as: decrease in test material, growth of microorganisms (fungi) and stability of the incubator temperature were also recorded. Once the time had elapsed, the test was completed, and the bioreactors were weighed. The test was performed in triplicate.

The calculation of the percentage of biodegradation was carried out using equations 3 and 4. The theoretical amount of carbon dioxide (ThCO_2_) evolved from the test material was obtained using Equation 3:


$$ThCO_{2}=m*wc*\frac{44}{12}$$ (3)

Where:

**m** is the mass of the test material placed in the container at the beginning of the test (g).

**wc** is the carbon contained in the test material, determined from the chemical formula or elemental analysis, expressed as a mass fraction.

The numbers **44** and **12** presented in equation 3 are the molecular masses of carbon dioxide and carbon, respectively.

The percentage of biodegradation (D_t_) for each test bioreactor (V_t_), from the carbon dioxide trapped during each measurement interval was obtained using Equation 4:


$$Dt=\frac{\Sigma {\left(CO_{2}\right)}_{T}^{t}-\Sigma {\left(CO_{2}\right)}_{B}^{t}}{ThCO_{2}}*100$$ (4)

Where:

$\Sigma {\left(CO_{2}\right)}_{T}^{t}$ is the accumulated amount of carbon dioxide, in grams, emitted by the test bioreactor (V_t_) between the beginning of the test and time t.

$\Sigma {\left(CO_{2}\right)}_{B}^{t}$ is the average cumulative amount of carbon dioxide, in grams, emitted in the blank containers (V_b_) between the beginning of the test and time t.

$ThCO_{2}$ is the theoretical amount of carbon dioxide, in grams, released by the test material.

Finally, according to the standard, to validate the results obtained in this test, the following points have to be considered: a) The reference material (microcrystalline cellulose) undergoes at least a 70% degradation after 45 days. b) The difference among the biodegradation percentages of the reference material in the different packaging is less than 20% at the end of the test. c) The inoculum in the blank container produces between 50 and 150 mg of CO_2_ per gram of volatile solids (average value) after 10 days of incubation.

### 2.8. Evaluation of ecotoxicity

The ecotoxicity of compostable plastics was evaluated with phytotoxicity tests. For this test, the methodology established in *OECD Test No. 208 Terrestrial plant Test: Seedling Emergence and Seedling Growth Test* [16]. For this purpose, seeds from: *Hordeum vulgare* and *Cynodon dactylon* were used.

The seeds of the aforementioned species were submitted to a viability analysis which was carried out in accordance with *OECD 208* [16]. Once the viability test was performed and after verifying that the tested seed lot showed germination greater than 70%, the phytotoxicity test was carried out. For this purpose, transparent polypropylene glasses with a capacity of 296 mL, the previously generated plastic-containing compost (mentioned in point 2.3.1), plastic-free compost, silica sand, microcrystalline cellulose, the seeds of the plant species, a thermometer-hygrometer, a scale with a sensitivity of 0.01 g and a homemade incubator for growing vegetables indoors were used.

25 g of plastic-containing compost and 25 g of silica sand were placed in each glass. Three seeds were placed in each glass for the test with barley, while 0.5 g of seeds were placed in each glass for the test with grass. Likewise, two control groups were established, one to which only 25 g of plastic-free compost (negative control) and 25 g of silica sand were added, and the other to which 25 g of plastic-free compost, 25 g of silica sand and 2 g of well mixed microcrystalline cellulose were added (positive control), and the same amount of seeds were placed in each control group. Subsequently, 35 g of distilled water were added with a sprinkler, and the glasses were placed in an incubator for indoor cultivation having 4 LED lights of 50 W with a spectrum of 400 to 500 nm and 650 to 700 nm, and two 20 W tube-type LED lamps with full spectrum (white light). Three repetitions were carried out per treatment.

Once the pots with the seeds were prepared, they were placed inside the incubator which was maintained at 22 °C (±5 °C) with a humidity content of 70% (±20%) and the photoperiod was adjusted at 16 hours of light and 8 hours of darkness with a timer. The test lasted for 21 days after the moment when 50% of the seeds in the control groups germinated. In the glasses containing barley, all the germinated seeds in each experimental unit were counted 7 days after the germination of the seeds in the control groups. The pots were watered by adding 20 mL of purified water every 3 days, and the status of the plants was checked every 7 days until they were 21 days old, recording any abnormal situation.

**Fig 4:**
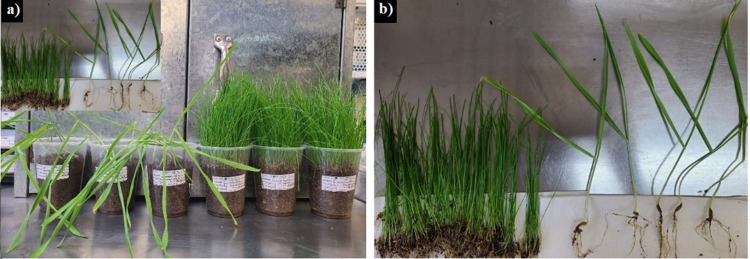
Example of *Hordeum vulgare* and *Cynodon dactylon* plants grown for 21 days for the evaluation of phytotoxicity. Left image, plants in substrate with compostable plastic. Right image, plant processing for measuring roots, height and biomass.

After 21 days, the plants were removed from the incubator and processed as follows: they were carefully removed from the glasses, the roots were cleaned with a little amount of water to remove soil particles and finally they were dried with paper towels. Subsequently, the height of the plants and the length of the root were measured, and then the plants were weighed to evaluate the wet biomass. The plants were then placed in an oven at 70 °C for 24 h to desiccate. Finally, they were weighed to evaluate the dry biomass. Fig 4 shows the processing of the plants.

### 2.9. Statistical analysis

The data analysis was carried out employing a design of a factor analyzed with an ANOVA test and a confidence of 95%, and subsequently a multiple range test (LSD).

## 3. Results and discussion

### 3.1. Analysis of the chemical composition of the bag

#### 3.1.1. Polymer analysis through ATR-FTIR spectroscopy.

**Fig 5:**
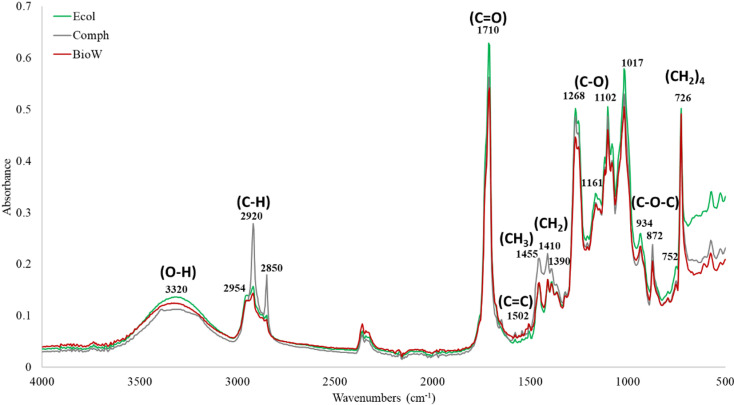
Infrarred spectrum of the compostable bag Ecol, Comph and BioW.

Fig 5 show the infrared spectra obtained from the different compostable bags tested. As can be seen in Fig 5, the infrared spectra of the three compostable bags are very similar since the highest and most representative peaks are observed in all of them. These results suggest that the three compostable bags contain the same polymers.

In the spectra of all the bags, there is a peak in the region 3300–3400 cm^−1^ characteristic of the OH group, two peaks in the region 2800–3000 cm^−1^ characteristic of the CH_3_ and CH_2_ group, which is reaffirmed since peaks are observed between the region 1475–1445 cm^−1^ and it is possible that there are chains of at least four CH_2_ since a peak is shown between 730–710 cm^−1^, likewise, a very important peak is found between 1710–1711 cm^−1^, characteristic of the carbonyl group. These functional groups are found in the molecular structure of PBAT, PLA, and corn starch, which suggests that they are present in the compostable bags under evaluation.

**Fig 6:**
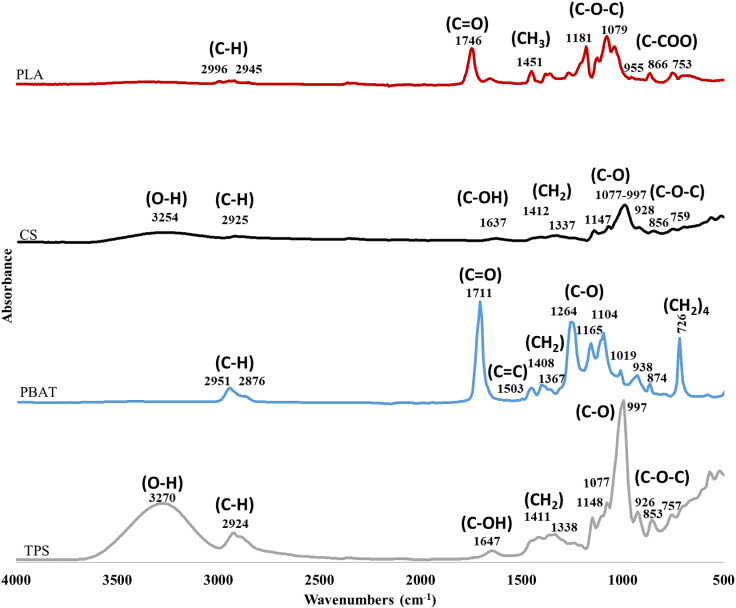
Infrared spectra of unmixed polylactic acid (PLA), corn starch (CS), polybutylene adipate-co-terephthalate (PBAT) and thermoplastic starch (TPS); the representative peaks of the spectra can be observed.

According to De Matos Costa [20], Weng [21], and Jian [22] the PBAT polymer that they reported showed peaks at 2960 and 2873 cm^−1^, an outstanding peak corresponding to the carbonyl group present in the molecule was also detected in the region 1700 and 1740 cm^−1^. Other reported important peaks were detected at 1504 cm^−1^, 1409 cm^−1^, 1395 cm^−1^, 1271 cm^−1^, 1104 cm^−1^, 1019 cm^−1^, 955 cm^−1^ and 731 cm^−1^. As can be seen in Fig 6, peaks at 1710–1711 cm^−1^, 1504–1506 cm^−1^, 1409–1410 cm^−1^, 1390 cm^−1^, 1267–1268 cm^−1^, 1101–1102 cm^−1^, 1016–1018 cm^−1^ and 726–727 cm^−1^ were detected in the spectra of the 3 compostable bags evaluated in the present study, which confirms that they contain the PBAT polymer.

As regards PLA, Alfei [23], Montañez-Supelano [24], Weng [21], Singla [25], Leroy [26], Chieng and [27] Thanki-Paragkumar [28], Yang [29] and Mofokeng [30] report that the characteristic peaks of PLA are found at 2996 cm^−1^, 2945 cm^−1^, 1757–1745 cm^−1^, 1457–1450 cm^−1^, 1425 cm^−1^, 1381–1386 cm^−1^, 1186–1190 cm^−1^,1160 cm^−1^ and 1083–1086 cm^−1^,1045 cm^−1^, 869 cm^−1^ and 757 cm^−1^.

As can be seen in Fig 5, the peaks at 2995–2998 cm^−1^, 2946–2947 cm^−1^ and 1745–1756 cm^−1^ characteristic of PLA according to the cited studies were not observed in any of the bags, which could indicate that it is not present in the bags. However, this polymer content is very low, which may explain the difficulty related to its detection. As regards this situation, in the present work, the IR spectra of an unmixed PLA, Corn starch (CS), PBAT, and Thermoplastic Starch (TPS) were obtained (Fig 6) against which the spectra of the tested bags were compared.

In Fig 6, the presence of the peaks mentioned by the different authors cited are present, emphasizing the peak at 1745.93 cm^−1^, representative of PLA. The signals close to 2919 cm^−1^ and 2849 cm^−1^ which were detected in the three bags tested cannot be observed. Although they can be related to the CH_3_ and CH_2_ groups, these peaks are not reported for any of the polymers that make up compostable bags according to the cited literature.

In some studies, where mixtures of PBAT and PLA were evaluated by FTIR, such as Al-Itry [31], Cardoso [32], Wu [33] and Rodrigues [34], the peaks related to C-H bonds in 2918–2920 cm^−1^ and 2849–2851 cm^−1^ were not reported either. On the other hand, it is also possible that these peaks are related to the portion of starch included in the tested bags being of the Thermoplastic Starch (TPS) type, since when comparing the IR spectra, with what was reported with Aversa and Barletta [35], show similarities.

In relation to the above, it cannot be ruled out that the peaks at 2918–2920 cm^−1^ and 2849–2851 cm^−1^ are related to the presence of polyethylene (PE), since according to Gulmine [36], Peltez and Simoneau [37], D’Amelia [38], Wang [39], Rodríguez-Bruceta [40], Sarker [41] and Bredács [42] the main signals detected in PE are: 2914–2920 cm^−1^ and 2847–2851 cm^−1^ (C-H bonds), 1450–1475 cm^−1^ (CH_2_ group) and 720–730 cm^−1^ (chains of four CH_2_).

Therefore, the signals 2918–2920 cm^−1^ and 2849–2851 cm^−1^ detected in all the compostable bags evaluated could be related to the presence of PE, TPS or some other additive, which is not clearly reported in the technique’s sheets, and that could be influencing the biodegradation performance of the bags (as reported later), mainly in the Comph bag, where the most intense signals were observed, and very low biodegradation performance.

Finally, according to Abdullah [43], Wang and Xie [44], Orsini and Aparicio [45] and Kizil [46], the spectrum of starch is between 3600–3300 cm^−1^, 2931–2928 cm^−1^, 1641–1637 cm^−1^, 1458–1458 cm^−1^ 1415–1412 cm^−1^, 1385–1375 cm^−1^, 1149 cm^−1^, 1200–800 cm^−1^, 930–920 cm^−1^, 856 cm^−1^ and 758 cm^−1^ for corn starch.

The presence of PBAT polymer was confirmed in the three bags evaluated at the time; however, it was not possible to establish its presence in relation to the presence of PLA. One explanation for the difficulty in identifying PLA in the analyzed bags may be its low content. To better clarify the possible presence of polymers in the samples, individual IR spectra were obtained for PLA, corn starch (CS), PBAT, and thermoplastic starch (TPS) (Fig 6).

As can be seen in Fig 6, in all the bags, peaks were found that could be related to PBAT and corn starch, and to a lesser extent to PLA. It is very important to highlight that the most easily identified polymer was PBAT. According to the data sheets, this polymer is found in a greater proportion in all the bags. It is also important to highlight the possible presence of PE or TPS.

#### 3.1.2. Analysis of metals through ICP-OES spectrometry.

The results obtained from the chemical analysis of metals in the compostable bags and their comparison with the maximum limits permitted by different regulations are shown in Table 2.

**Table 2:** Average amount (mg/kg) of metals detected in compostable bags and its comparison with the maximum permissible limits (MPL) accord with NOM-004-SEMARNAT-2002^1^, UNE-EN 13432:2001^2^ and ASTM D6400-23:2020^3^.

Element	As	Cd	Co	Cr	Cu	Mo	Ni	Pb	Se	Zn
λ (nm)	**188.98**	**226.502**	**228.615**	**267.716**	**324.754**	**202.032**	**231.604**	**220.353**	**196.026**	**213.857**
	**(mg/kg)**									
MPL Excellent^1^	41.0	39.0	n/a	1200.0	1500.0	n/a	420.0	300.0	n/a	2800.0
MPL Good^1^	75.0	85.0	n/a	3000.0	4300.0	n/a	420.0	840.0	n/a	7500.0
MPL^2^	5	0.5	n/a	50	50	1	25	50	n/a	150
MPL^3^	20.5	19.5	n/a	n/a	750	n/a	210	150	50	1400
BioW	2.5	0.5	2.5	7.7	46.5	1.0	9.0	2.5	2.5	9.0
Comph	2.5	0.5	2.5	3.2	3.0	0.5	0.7	9.2	2.5	76.6
Ecol	2.5	0.5	2.5	1.3	2.8	0.5	0.8	2.5	2.5	50.0

As can be seen in Table 2, none of the compostable bags exceed the maximum permissible limit of metals established by *NOM-004-SEMARNAT-2002*, *UNE EN 13432:2000* and *ASTM D 6400* [17–19]. It is important to highlight that some of the metals detected, such as Zn, Mo, Cu and Ni, are considered micronutrients necessary for the correct development of plants. It can also be confirmed that the Mexican standard is more tolerant of the metal content in compostable bags.

Likewise, in Table 2 it can be seen that there were differences between some metals contained in each bag. In the case of Zn, a very variable amount is observed between the 3 bags, ranging from 76.6 mg/kg in Comph, to 50 mg/kg in Ecol and 9 mg/kg in BioW. Regarding Pb, a content of 9.2 mg/kg is observed in the Ecol bag, being the highest compared to the other two bags. And finally, regarding Cu, Ni and Cr, it is observed that only the BioW bag has a high content compared to the other 2 bags, with values of 46.5 mg/kg, 9 mg/kg, and 7.7 mg/kg, respectively. It is important to mention that, according to Kaiser [47], the higher content of Cu, Ni and Co in compostable bags compared to the other metals, is mainly due to the use of these metals as pigments to give green, yellow and blue hues to the polymers during the production of the bag and as ink for printing on them. The above is consistent with the results obtained, since a characteristic of the BioW bag is its green color.

As for the other metals (Zn and Pb), the higher quantities may be mainly due to the quality of the polymers used; when comparing the quantity of these metals with those of the other authors, no major differences in their content were observed. In works such as those by Gómez [48], De Fuentes [49] and Huerta-Pujol [50] where the metal content in compostable bags made with PBAT and PLA was also verified to ensure regulatory compliance and the safety of commercial bags, it was found that all cited works show results similar to those observed in this study, and that these products tend to be within the LMP established by UNE EN 13432:2000.

### 3.2. Disintegration, aerobic biodegradation and ecotoxicity

#### 3.2.1. Percentage of disintegration (D) in accordance with *ISO 20200:2015 standard.*

**Fig 7:**
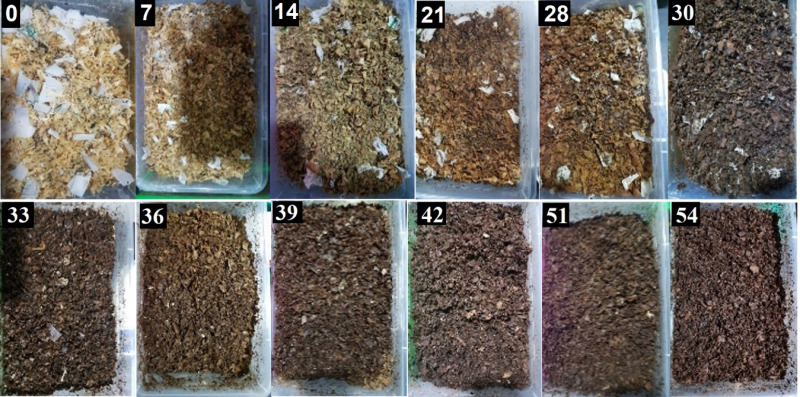
Example of the evolution of the bioreactor with compostable plastic bags.

The results obtained for this test, shown in Tables 3 and 4, give the validation results of the composting process for each bioreactor, and the evolution of the visual appearance of the bioreactors can be seen in Fig 7.

**Table 3:** Average percentage of disintegration (D) and percentage of recovery of bags.

Bioreactor	Home weight (g)	End weigth (g)	Desintegration (D) (%)	Desvest	Recovery (%)	F value	p value (α_0.05_)	LSD
BioW	15	3.503	76.64	0.5	23.36	2276.36	4.07	8.62
Comph	15	0.613	95.91	1.9	4.09	–	–	–
Ecol	15	1.387	90.76	2.5	9.24	–	–	–
CT-	15	15	0.00	0.0	100.00	–	–	–

CT- black polypropylene bag.

**Table 4:** Average percentage of the validation of decrease in volatile solids (R) in the bioreactors.

Bioreactor/Repetition	Value R
BioW	43.0
Comph	44.9
Ecol	41.7
CT-	43.06

As can be seen in Table 3, out of the three bags tested to evaluate the percentage of disintegration, only two passed the test, these being the Comph bag that showed a D value of 95.91% on average, and the Ecol bag with a D value of 90.75% on average. In contrast, the BioW bag showed a D value of 76.64% on average, and finally the negative control bag made of polypropylene (CT-) showed a D value of 0%, which was expected. Likewise, when performing the analysis of variance (ANOVA), significant differences were observed between the disintegration rate (D) of the bags tested. Subsequently, the LSD test showed that there is a significant difference between the mean of CT- and the mean of all compostable bags (expected result). Likewise, significant differences were obtained between the BioW bag and the Comph and Ecol bags, and finally no significant differences were shown between the Comph and Ecol bags.

As regards the validity of the results, Table 4 shows the results of the validation of the composting process of each bioreactor tested. Using the equation for the decrease in volatile solids (R), it was found that all bioreactors exceeded R 30%, which is the minimum acceptable value. The bioreactors were between 37% and 46%. According to *ISO 20200:2015* [14] an average R value for this test is 42.3%.

The results obtained in this test seem to follow the same trend as those reported by Vaverkova [51], Intaraksa [52], Sarasa [53] y Arrieta [54] Siriyota [55] who, using the same methodology described in *ISO 20200:2015* standard [14], reported a disintegration of 63.6.00 to 100.00% for bags made of compostable polymers, such as starch, PBAT, PCL, PLA, PHB and mixtures of these.

On the other hand, Siriyota [55] obtained a disintegration of 25.91% with the non-mixed ECOFLEX polymer and 56.29% with the non-mixed EMPOL polymer, which indicates that the starch content greatly influences the disintegration rate. In relation to this, Gattin [56] mentions that in some studies it has been shown that the content of starch in a mixture with other biodegradable polymers helps disintegration, because starch is a highly biodegradable polymer, and its biodegradation can stimulate the fragmentation of the other polymers. Similarly, Nandakumar [57], indicates that during degradation, mixtures of polymers with starch can weaken due to the activity of microbes in the starch portion which generally leads to biofragmentation.

According to the previous paragraph, it is observed that the results obtained in this study are consistent with the results of other studies, and that it was also possible to observe differences in the disintegration percentages for each bag tested, this situation may be directly related to the proportions of each polymer that composes them. Chinaglia [58] mentioned that the biofragmentation of polymers seems to depend on the surface area of the polymer, since they observed that this can limit degradation in most cases, the greater the surface area the greater the degradation. This situation is attributable to the fact that the degradation of these materials is mainly caused by enzymes, and the enzymes are limited by the size of the surface. Likewise, Jiang [59] suggests that the degradation rate depends on some factors such as the thickness of the material, the composting conditions and the chemical composition of the material, and many possibilities can be found by modifying these factors.

#### 3.2.2. Percentage of aerobic biodegradation (D _t_ ) in accordance with *ISO 14855:2018-2* standard.

The results obtained as regards the characterization of the inoculum that was used for the aerobic biodegradation test are shown in Table 5. Likewise, the results of total organic carbon (TOC) contained in the different bags and reference material (control) are shown in Table 6.

**Table 5:** Average dry mass, volatile solids and pH determined in the inoculum used in the aerobic biodegradation test.

Sample (g)	Water weight (g)	Dry mass (g)	Dry mass (%)	Volatile solids (g)	Volatile solids (%)	pH
10	5.375	4.625	46.253	1.6052	34.705	7.893

**Table 6:** Average total, organic and inorganic carbon present in the compostable bags and the reference material.

Bag/Material	Total organic carbon (COT %)	Inorganic carbon (CI %)	Total carbon (CT %)
BioW	53.85	0.09	53.95
Comph	34.87	0.02	34.89
Ecol	34.38	0.00	34.38
CT	25.71	0.00	25.71

CT microcrystalline cellulose control.

As can be seen in Table 5, the inoculum used for the aerobic biodegradation test met the characteristics established by the *ISO 14855:2018-2* standard [15], which indicates that the inoculum must contain between 35 and 55% dry mass, the volatile solids content must be 30% or greater in the dry mass previously obtained, and the pH of the inoculum must be between 7.0 and 9.0. On the other hand, according to Table 6, the bag that presented the highest COT value was BioW with 53.85% on average, followed by Comph with 34.87% and Ecol with 34.38%.

**Fig 8:**
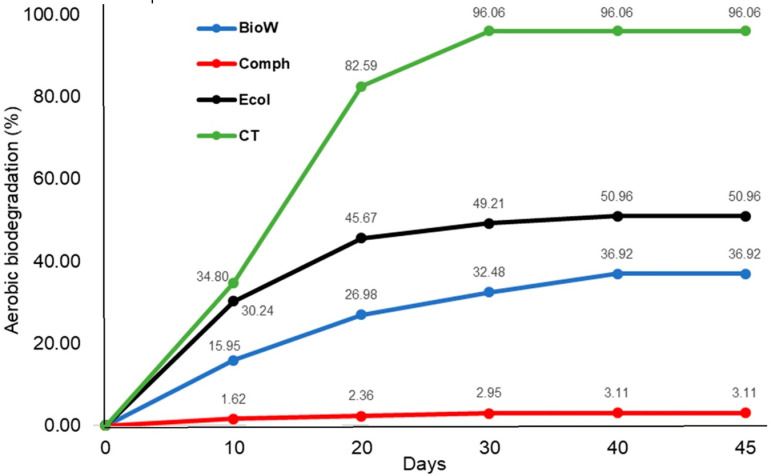
Percentage of average aerobic biodegradation with respect to time (days).

The results of the average biodegradation percentage (D_t_) obtained in the present study are shown in Fig 8.

As can be seen in Fig 8, of the three bags that were tested to evaluate the percentage of aerobic biodegradation, none achieved the minimum value required to pass the test, which is 90% as regards the calculated theoretical CO_2_. The bag that obtained the highest percentage on average was Ecol with a D_t_ of 51% ±  7.73, followed by BioW with a D_t_ of 36.92% ±  5.23, and finally Comph that showed a D_t_ of 3.11% ±  1.50 on average. In contrast, the control group with the reference material CT showed an aerobic biodegradation of 96.06% ±  2.99 on average, this being a positive indicator of the assembly and conduction of the test. Likewise, when observing Fig 8, it can be seen that the time interval is around 20 to 30 days to obtain maximum aerobic biodegradation in all treatments including the control group.

When performing the statistical analysis (ANOVA), indicating that there are significant differences between the aerobic biodegradation rate (D_t_) of the different bags tested, including the control group. Subsequently, the LSD indicated that there is a significant difference between the mean of CT and the mean of all compostable bags. Likewise, significant differences were obtained between the Comph bag and the Ecol and BioW bags, and finally no significant differences were shown between the BioW and Ecol bags.

The results obtained indicate that none of the bags tested can be considered biodegradable according to the established criteria. However, it can be mentioned that the Ecol and BioW bags are partially biodegradable since a biodegradation percentage close to 50% in relation to total organic carbon was shown. On the other hand, the Comph bag could be considered non-biodegradable since the value obtained was very low. These results could be related to the possible presence of PE or some additive in the bags, since the Comph bag was the one that presented the highest intensity in the characteristic signals of this polymer (see Fig 6).

The method used to determine the final aerobic biodegradation was set out in ISO 14855:2018-2 [15]. This method establishes controlled composting conditions to obtain the final aerobic biodegradability % value by gravimetric measurement of the amount of carbon dioxide released. The results were expressed as the final aerobic biodegradability. Fig 8 shows the evolution of this biodegradability process over time to reach practically constant values between two continuous measurements. The development process under the best biodegradability conditions was confirmed with the values obtained for aerobic biodegradability with the control sample (microcrystalline cellulose) reaching in excess of 96% after 30 days.

As regards the validation of the results obtained in this test, it is highlighted that the control group showed a Dt of 90% at 30 days which fulfills the first condition (section a). Likewise, no differences greater than 20% were observed in the biodegradation percentages of the three bioreactors that make up the control group, which complies with the second condition (section b). Finally, the blank bioreactors generated 169.2 mg (Blank 1), 136.3 mg (Blank 2), and 125.78 mg (Blank 3) of CO_2_, averaging 143.76 mg in the first 10 days of the test, which is within the expected range and fulfills the condition set up in section c, so the test was deemed successfully passed.

When comparing the results obtained in the present study with the results of some studies that used similar methodologies for the evaluation of the percentage of biodegradation on pure polymers or mixtures of compostable/biodegradable polymers, very diverse results were observed, which were similar or very different from those reported. However, consistency was shown in the percentage of biodegradation achieved by microcrystalline cellulose (control). As an example, can mention the work carried out by Tabasi and Ajji [60], who evaluated the percentage of aerobic biodegradation of PLA and PBAT reporting that the. The biodegradation percentage of their control group was 93%.

Dammak [61] evaluated the biodegradation of mixtures of PBAT with thermoplastic starch (TPS). His results showed a percentage of biodegradation of TPS and PBAT in pure form reached a value greater than 90% in 90 days. Likewise, the reference material used (microcrystalline cellulose) also achieved a biodegradation greater than 90%.

Petinakis [62], using *ISO 14855* standard [15], reported biodegradation was less than 60% for pure PLA, 80% for the first mixture, 60% for the second mixture and 50% for the third mixture. In this study, a biodegradation of microcrystalline cellulose greater than 70% in 40 days was also reportedand Cadar [63] and Ahn [64] reported thatcontrol (microcrystalline cellulose) showed a biodegradation percentage of 76%.

Other results shown by Ruggero [65] and Emadian [7] who conducted a review of different studies where the percentage of biodegradation of different polymers was evaluated in accordance with *ISO 14855* standard [14] and other similar methodologies, report that Kale [66] obtained a PLA biodegradation percentage of 80% in a 60-day test, Iovino [67] obtained a biodegradation percentage of 87% in a 90-day test carried out on starch-based polymers, and Du [68] obtained a biodegradation percentage of 70% also with starch-based materials.

On the other hand, other studies such as that of García-Depraect [12], where aerobic biodegradation was analyzed in accordance with *ISO 14852* standard, the results showed that after 117 days the percentage of biodegradation of plastics based on PHB, PHBV and PCL were 79.6%, 84.5% and 75.7%, respectively. On the other hand, the biodegradation percentage of PBS, PBAT, PLA and a PLA-PCL mixture was less than 10%. This study also reported a biodegradation of microcrystalline cellulose of 86.8%. These results contrast with the BPAT biodegradability percentage reported by Dammak [61], while they agree with Tabasi and Ajji [60], for PBAT and with Ahn [64], for PLA.

As can be seen in all the cited studies, the proportions of each polymer in the different mixtures, as well as the methodology used to manufacture them, can influence their final biodegradation percentage; which is coherent and could possibly explain the different biodegradation percentages obtained in the present study. In agreement with this, Abe [69] reports that mixtures in a polymeric bioplastic matrix influence biodegradation (increasing or reducing biodegradation), since the different components of plastic materials can influence the accessibility of enzymes to the polymeric material in different ways. Likewise, it is important to mention that it is difficult for the biodegradation of some biodegradable natural polymers to reach 100% in a few months since it has been reported that this can take longer depending on the conditions. For example, in some studies it has been observed that although starch is a 100% biodegradable polymer, its total biodegradation can take up to 1 year. Likewise, the biodegradability of some polymers does not reach 100% because they can be used for the synthesis of polysaccharides necessary for microorganisms, which prevents its total mineralization (partial mineralization), this being the case of starch, a component of the compostable bags evaluated in the present study [70].

In addition to the above, and in relation to the different proportions of polymers used for the production of compostable bags tested in this study, German [71] reported that the rate of starch biodegradation depends on the concentration, and that this can decrease by up to 50% when the starch present is < 10% of the soil organic matter. This reduction in biodegradation is attributed to a decrease in enzymatic activity, since the energy costs for microorganisms to produce extracellular enzymes are too high compared to the metabolic energy yield when little starch is available. This phenomenon plus the evidence that starch mixed with other biodegradable polymers, such as PLA and PBAT, helps the fragmentation and biodegradation of the latter may be related to the results obtained, with the starch content in each bag being an important factor. Likewise, there seems to be an influence of the method used to determine biodegradation as well as the physical and chemical characteristics of each polymer, with the percentage of biodegradation obtained at the end of a test.

#### 3.2.3. Ecotoxicity according to *OEDC 208* standard.

The results obtained from the survival, root size, stem size (aerial part), wet biomass and dry biomass of the plant species used are shown in Tables 7 and 8. It is important to mention that the *H. vulgare* seeds reached a 100% germination. Likewise, the survival of the plants after 21 days of cultivation was 100%. In the case of *C. dactylon*, no germination count was carried out since 0.5 g of seeds were added, nor was a survival count required since this is evaluated through biomass measurements.

**Table 7:** Average of measurements made on stems, roots and biomass of *H. vulgare* plants grown in compost with different compostable plastics and silica sand (50/50 mixture).

Treatment	Steam (cm)	Root (cm)	Total (cm)	Wet biomass (g)	Dry biomass (g)
BioW	28.38	13.17	41.54	0.54	0.07
Comph	29.09	12.73	41.82	0.53	0.06
Ecol	27.31	11.16	38.47	0.59	0.06
C−	27.91	13.91	41.82	0.61	0.08
C+	27.92	14.33	42.26	0.70	0.08
F value	0.17	1.16		2.03	3.48
p value (α_0.05_)	3.48				

C +, control compost with microcrystalline cellulose; C−, compost alone.

**Table 8:** Average of measurements made on stems, roots, and biomass of *C. dactylon* plants grown in compost with different compostable plastics and silica sand (50/50 mixture).

Treatment	Stem (cm)	Root (cm)	Total (cm)	Wet biomass (g)	Dry biomass (g)
BioW	15.87	2.60	18.47	3.19	0.38
Comph	16.07	2.63	18.70	3.28	0.41
Ecol	15.83	2.73	18.57	3.14	0.42
C-	16.60	2.90	19.50	3.33	0.42
C+	15.97	2.73	18.70	3.37	0.42
F value	1.67	2.93		1.62	0.33
p value (α_0.05_)	3.48				

As can be seen in Table 7, the *H. vulgare* plants grown in compost from Comph bags had on average the greatest stem length (leaf length), followed by the BioW treatment, negative control (C-) and positive control (C+) and, finally, the Ecol treatment. The plants in the C + group had, on average, the greatest root length, followed by the C− group, BioW treatment, Comph treatment and finally the Ecol treatment. As regards wet biomass, the C+ group had the highest amount on average, followed by the C− group, Ecol treatment, BioW treatment and the Comph treatment. Finally, in terms of dry biomass, the plants grown in C + generated on average the highest amount of dry biomass, followed by the C− group, BioW treatment, Ecol treatment and finally the Comph treatment.

When performing the analysis of variance, it was observed that there were no significant differences between the treatments. These results suggest that there is no phytotoxic effect of the compostable plastics tested on *H. vulgare*.

Table 8 shows the results obtained with the cultivation of *C. dactylon.* The grass grown in the C− group had, on average, the greatest stem length (leaf length), followed by the Comph treatment, then the C+ group, the BioW treatment and finally the Ecol treatment. The grass in the C− group had, on average, the greatest root length, followed by the C+ group and the Ecol treatment, then the Comph treatment, and finally the BioW treatment. As regards wet biomass, C+ group had the highest amount, on average, followed by C− group, Comph treatment, BioW treatment and Ecol treatment. Finally, in terms of dry biomass, the plants grown in the C+ group, C− group and Ecol treatment had the highest amount, on average, followed by the Comph group and finally the BioW group.

In the same way as with *H. vulgare*, when performing the analysis of variance, it was observed that there were no significant differences between the treatments. These results suggest that there is no phytotoxic effect of the compostable plastics tested on *C. dactylon*

The results obtained in the present study agree with those reported by Palsikowski [72], Rudeekit [73] Greene [74], Álvarez [75], Liwarska-Bizukojc [76,77], Barbale [78] and Gómez [48] who evaluated the phytotoxicity of PBAT, PLA, starch and mixtures of these polymers on different vegetal species such as *Allium cepa, Oryza sativa, Vigna radiate, Physalis ixocarpa*, *Sorghum saccharatum*, *Lepidium sativum*, *Sinapsis alba Nasturtium officinale*, *Hordeum vulgare* and *Lactuca sativa* no toxic effect of the polymers on the plants was observed.

## 4. Conclusions

According to the results obtained in the present study, it was observed that the polymers Polybutylene adipate-co-terephthalate (PBAT) and corn starch were detected in the three compostable bags evaluated, confirming the information stated in their data sheets. However, the detection of Polylactic Acid (PLA) was not so evident, which may be directly related to the fact that it is the polymer with the lowest reported content in the three bags, and there is the possibility that the bags tested may contain low amounts of polyethylene (PE), or some additive, or may contain termoplastic starch (TPS).

All compostable bags tested were found within the maximum permissible limits in mg/kg of metal content (As <  5, Cd <  0.5, Cr <  50, Cu <  50, Mo <  1, Ni <  25, Pb <  50, Se <  50 and Zn <  150) in accordance with national and international regulations, complying with the chemical composition criteria established for compostable plastics.

The high quantities of metals such as Cu, Ni and Co detected in compostable plastic bags are related to the use of pigments of mineral origin (inorganic).

Comph and Ecol compostable bags passed the standardized test for assessing disintegration (fragmentation), suggesting that a satisfactory percentage of disintegration is not directly related to a high percentage of biodegradation.

None of the tested bags passed the chemical degradation criterion (percentage of biodegradation through CO_2_ capture), however, important differences were observed between Ecol (50.96%) and BioW (36.92%) compared to Comph (3.11%). It is possible that the biodegradation performance of the bags is related to the presence of polyethylene in low quantities.

None of the bags evaluated showed a toxic effect on *H. vulgare* and *C. dactylon*, however, it is important to consider other groups of model organisms to evaluate the toxicity of these materials.

The evaluated plastic bags made with PBAT, PLA and corn starch partially covered the requirements established for compostable plastics, however, they cannot be considered one hundred percent compostable. It is necessary to standardize the manufacturing process of compostable bags in terms of the proportions of polymers that compose them and their physical characteristics, since this has a direct impact on the performance of the materials to meet the criteria that are evaluated to certify the compostability of a plastic bag.
